# Association of Subregional Quantitative Ultra-widefield Fluorescence Angiography Characteristics With the Occurrence of Diabetic Macular Edema and Proliferative Diabetic Retinopathy

**DOI:** 10.3389/fmed.2021.720564

**Published:** 2021-09-24

**Authors:** Gongpeng Sun, Xiaoling Wang, Jingwen Jiang, Zuohuizi Yi, Mei Fu, Xueying Yang, Hongmei Zheng, Changzheng Chen

**Affiliations:** Eye Center, Renmin Hospital of Wuhan University, Wuhan, China

**Keywords:** ultra-widefield fluorescein angiography (UWFFA), diabetic macular edema (DME), proliferative diabetic retinopathy (PDR), leakage index, ischemic index, microaneurysm count

## Abstract

**Purpose:** To explore the relationships of region-specific properties of ultra-widefield fluorescence angiography (UWFFA) images with two adverse outcomes, diabetic macular edema (DME) and proliferative diabetic retinopathy (PDR), and also the severity of diabetic retinopathy (DR).

**Methods:** A cross-sectional observational study was performed to retrospectively analyze UWFFA images of patients with DR. All patients underwent UWFFA and optical coherence tomography examination. Leakage index and microaneurysm (MA) count were measured using Trainable Weka Segmentation, a machine learning algorithm, and ischemic index (ISI) was measured manually. The correlation between UWFFA parameters and severity of DR was analyzed, and receiver operating characteristic curves were used to estimate their diagnostic value for DME and PDR.

**Results:** A total of 108 eyes from 108 patients with DR (mean age of 56.04 ± 8.85 years) were analyzed. As the severity of DR increased, the ISI and leakage index of the panretina and all subregions increased. Panretinal MA count and leakage index were significantly higher in eyes with DME than those without DME (*p* = 0.044 and 0.001, respectively). Leakage index and ISI were significantly higher in eyes with PDR than those without PDR in both panretinal and subregion-specific measurements (all *p* < 0.05). Throughout the retina and specifically in the posterior area (PoA), the leakage index had a higher diagnostic value for DME than ISI or MA count (all *p* < 0.05). The diagnostic value of MA count for PDR was lower than that of ISI and leakage index (all *p* < 0.05).

**Conclusion:** The ISI, leakage index, and MA count in the PoA and panretina correlated with the severity of DR, especially the posterior parameter. The leakage index was more valuable than ISI and MA count in determining the occurrence of DME. ISI and leakage index were better predictors of PDR.

## Introduction

Diabetic retinopathy (DR) is one of the leading causes of vision loss and blindness and has an increasing prevalence in the working-age population worldwide from 1990 to 2020 (1). The number of patients with diabetes is expected to exceed 600 million by 2040, while DR and resulting visual impairment are becoming increasingly prevalent as people with diabetes live longer (2, 3). Diabetic macular edema (DME) and proliferative diabetic retinopathy (PDR) are severe vision-threatening endpoints in patients with DR. DR, PDR, and DME affect 34.6, 7.0, and 6.8% of patients with diabetes, respectively (3). Therefore, predicting the occurrence of DME and PDR is critical.

The pathogenesis and risk factors for DME and retinal neovascularization are complex and diverse. Fundus fluorescence angiography (FFA) is an important imaging tool for assessing the vasculopathy of DR. The range of conventional FFA is 30–55° (4, 5), and 7 Standard Field (7SF) imaging after montage has only a 75° imaging range. Ultra-widefield fluorescence angiography (UWFFA) can expand this range to about 3.2 times that of 7SF (5), achieving a 200° range in one image, allowing clinicians to better observe changes in the diabetic retinal peripheral fundus, especially in the peripheral nonperfusion area and neovascularization. UWFFA can help visualize more retinal vascular lesions in the fundus of patients with DR compared to conventional FFA (5–8).

To more accurately assess the severity of DR and its complications, a series of imaging parameters, namely, ischemic index (ISI), leakage index, microaneurysm (MA) count, retinal vascular bed area, and fractal dimension, have been established and applied to the analysis of UWFFA images (9–15). Moreover, ischemia, leakage, and MA are the most characteristic imaging changes on FFA in patients with DR. Ehlers et al. (16) first reported that the panretinal ISI, leakage index, and MA count correlate with the severity of DR and noted that the posterior leakage index and MA count correlate with the presence and severity of DME.

Different regions of the retina have different distributions of photoreceptor cells and different metabolic capacities (17). It is therefore critical to study region-specific changes in the retina with ultra-widefield (UWF) imaging. In this study, we partitioned the distribution of MA count, ISI, and leakage index in UWFFA images of patients with DR with different levels of disease severity and systematically evaluated the correlation of these parameters with DME and PDR.

## Methods

### Study Participants

This is a cross-sectional observational study that retrospectively analyzed UWFFA images of patients with DR who visited the Renmin Hospital of Wuhan University from June 2016 to October 2020. This study was reviewed and approved by the Clinical Research Ethics Committee of Renmin Hospital of Wuhan University (approval number: WDRY2020-K034). Given the retrospective nature of this study, an application for an informed consent waiver for patients was submitted to and approved by the ethics committee. All the involved patients underwent slit-lamp examination, UWF fundus color photography, UWFFA, and optical coherence tomography (OCT).

### Inclusion and Exclusion Criteria

Inclusion criteria were: age > 18 years; diagnosis of DR (namely, type 1 and type 2 diabetes). Exclusion criteria were: previous retinal photocoagulation and vitrectomy; antivascular endothelial growth factor (anti-VEGF) treatment within 3 months; artifacts in the UWFFA image that significantly affect the evaluation of macular edema and all parameters (such as eyelashes, eyelids, and gloves of the person who took the image); poor-quality OCT images or OCT images not centered on the macula; severe media opacity (such as cataract and vitreous hemorrhage); combined retinal vein obstruction, uveitis, and macular degeneration that may cause macular edema; optical coherence tomography image of the epiretinal membranes or vitreous macular traction and other diseases that may cause retinal thickening; the presence of tractional retinal detachment; and high myopia.

### Image Acquisition

**Figure 1:**
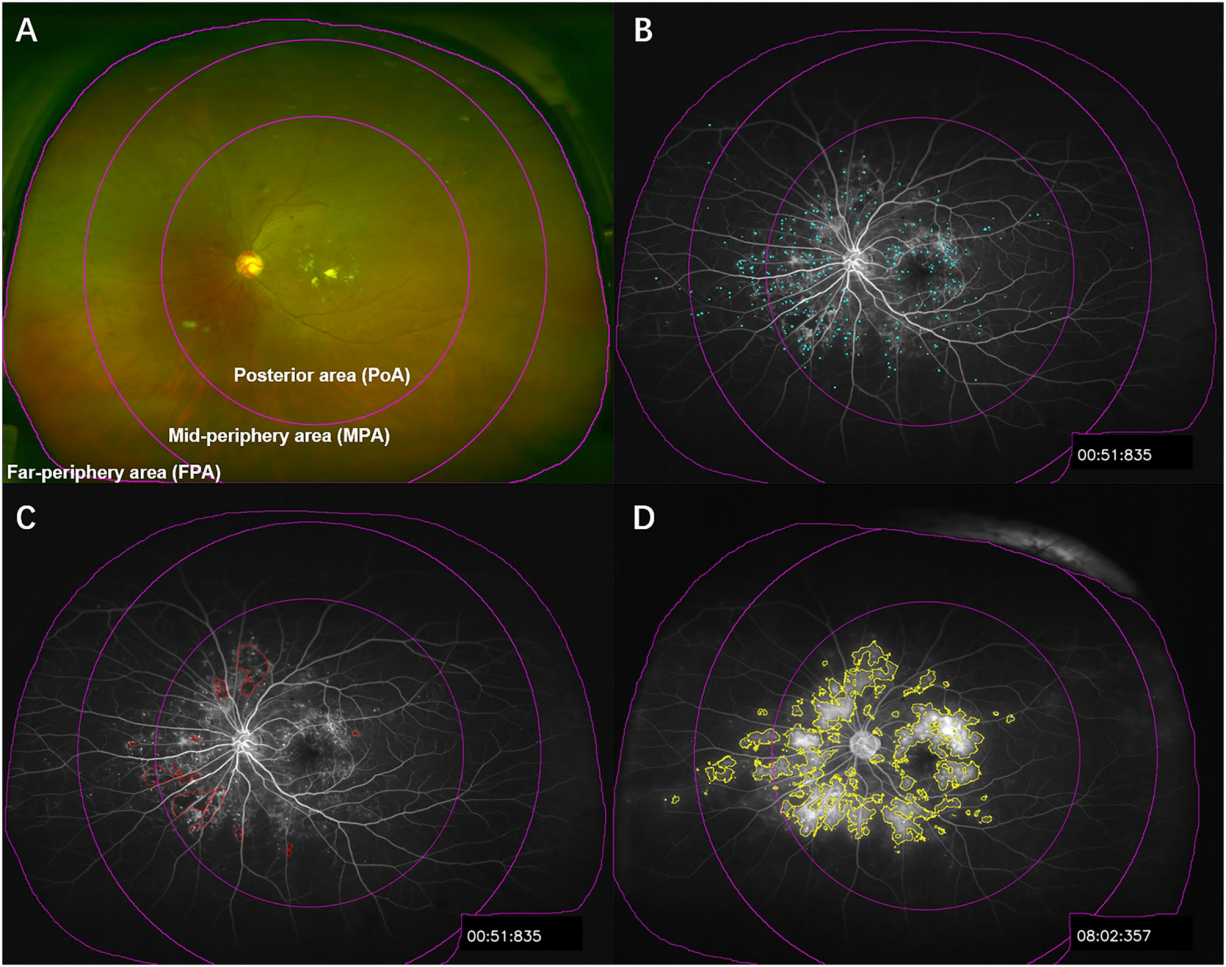
Illustration of UWFFA partitioning and segmentation of microaneurysm, ischemia, and leakage. **(A)** The UWF pseudocolor image of patients with DR was divided into panretina, posterior area, mid-periphery area, and far-periphery area, corresponding to the subdivisions in the UWFFA images. **(B–D)** Examples of microaneurysm, ischemia, and leakage segmentation, in sequence.

All UWF pseudocolor images and UWFFA images were obtained by Optos 200Tx or California (Optos plc, Dunfermline, UK) by the two experienced physicians. Briefly, the UWF pseudocolor images were first obtained after the pupil of patients was dilated (Figure 1A). After intravenous injection of 5 ml of 10% sodium fluorescein, macula-centered UWFFA images and guided eye images of the patients in the superior, inferior, nasal, and temporal directions were obtained within 0–10 min. Macula-centered images from 45 s to 1 min and 30 s (early phase) and macula-centered images from 5 to 10 min (late phase) were selected to calculate UWFFA parameters, respectively. The eyes with a wider field of view and a clearer UWFFA image were selected for analysis.

The average retinal thickness in the 1 mm range of the macula and the total retinal volume in the 1, 3, and 5 mm ranges centered on the macula were measured with RTVue XR Avanti (Optovue Inc., Fremont, CA, USA).

### Image Processing

Images were processed using the public domain software Fiji (http://fiji.sc/Fiji). To speed up image processing, we first transformed the UWFFA images by bilinear interpolation from 3,900 × 3,072 pixels to a size of 1,170 × 922 pixels where the details were still distinct (18).

MA count and leakage index were obtained by Trainable Weka Segmentation, a machine learning algorithm (Figures 1B,D). The training features were Gaussian blur, Hessian, membrane, Sobel filters, and difference of Gaussians, and the classifier was FastRandomForest, a multithreaded version of random forest initialized with 200 trees and two random features per node. Images with distinct features were selected for training to obtain satisfactory extraction results. MA segmentation was achieved by analyzing early UWFFA images with a trained algorithm, and MA count was obtained using the Analyze Particles tool. The leakage index was obtained by analyzing the late UWFFA images. Since the brightness and contrast of some leakage regions were relatively low, we first applied the Enhance Local Contrast tool, and then used this preprocessed image to train the algorithm and segment the leakage area. If there was no significant edema or fluorescent leakage from the optic disc, it would be manually excluded from the leakage area.

Given that nonperfusion area (NPA) is easily affected by hyperfluorescence, such as vascular filling or vascular leakage, and that peripheral background hypofluorescence is high in some patients, the segmentation of the NPA was difficult to achieve by similar automated algorithms. We, therefore, opted for a more reliable manual selection method (Figure 1C). By referring to the superior, inferior, nasal, and temporal UWFFA images in the middle phase, image processors delineated the NPA in the early phase.

Segmentation of ischemia, leakage, and MA was performed by a trained medical student, and the resulted segmented images were overlayed on the corresponding original images. The synthesized resultant images were reviewed by an ophthalmologist with extensive experience working with UWFFA images. For images with unsatisfactory segmentation, manual adjustment of the feature segmentation was directed by a fundus specialist. To avoid subjective factors affecting the image processing results, the retinal thickness of the patients was masked when extracting the UWFFA parameters.

The severity of DR was graded according to the Diabetic Retinopathy Disease Severity Scale using the corresponding UWF pseudocolor images (19). The DME is defined as the mean retinal thickness within 1 mm of the macula >300 μm or the presence of subretinal fluid within it. Leakage index is defined as the ratio of leakage area to the total area of the corresponding retina, ISI is defined as the percentage of the NPA to the total area of the corresponding retina, and MA count is defined as the total number of MAs within a certain retinal area.

To investigate the relationship between various parameters in different regions on two adverse outcomes, DME and PDR, we divided the images into three regions centered on the macula (Figure 1A), namely, the posterior area (PoA; 0–10 mm), the mid-periphery area (MPA; 10–15 mm), and the far-periphery area (FPA; 15 mm to the visible retinal boundary), based on the method of Silva et al. (8). Different regions were selected with the Region of Interest manager and the Specify tool.

### Statistical Analysis

The normality of the data was tested by Shapiro–Wilk test and histograms. Due to the relatively small sample size, the quantitative parameters in this study were nonnormally distributed and expressed as median and 95% CI. Regional UWFFA parameters in patients with different severity levels of DR were compared using the Kruskal–Wallis H test. Correlations between UWFFA parameters and the severity of DR were analyzed using multivariate ordered logistic regression. The differences in UWFFA parameters in different regions between eyes with and without DME were determined by the Mann–Whitney U test. The discriminative capacity of the three UWFFA parameters in different regions for DME and PDR was evaluated by receiver operating characteristic (ROC) curves and the area under the ROC curve (AUC), and the optimal cut-off values were identified. Multiple linear regression analysis was used to analyze the correlation between the UWFFA parameters and central macular thickness (CMT) or central macular volume (CMV). All data processing was performed using SPSS 20.0 (version 20.0; SPSS, Chicago, IL, USA), and a *p* < 0.05 was considered statistically significant.

## Results

A total of 108 eyes from 108 patients with DR with a mean age of 56.04 ± 8.85 years were analyzed in this study, namely, 51 (47.22%) females and 54 (50%) right eyes. There were 11 eyes with mild nonproliferative diabetic retinopathy (NPDR), 29 eyes with moderate NPDR, 25 eyes with severe NPDR, and 43 eyes with PDR. A total of 36 (33.33%) eyes were diagnosed with DME. The demographic and clinical characteristics of patients with different severity levels of DR are listed in Table 1.

**Table 1:** Demographical and clinical characteristics of the studied eyes with diabetic retinopathy.

**Characteristic**	**Mild NPDR (*n* = 11)**	**Moderate NPDR (*n* = 29)**	**Severe NPDR (*n* = 25)**	**PDR (*n* = 43)**	***p-*value**
Age, y	50.00 (46.00–65.00)	58.00 (56.00–63.50)	61.00 (56.00–64.00)	55.00 (51.00–57.00)	**0.003** ^*^
Female, *n* (%)	6 (54.54%)	14 (48.28%)	12 (48%)	19 (44.19%)	0.937^†^
Right eye, *n* (%)	5 (45.45%)	14 (48.28%)	15 (60%)	20 (46.51%)	0.721^†^
DME, *n* (%)	0 (0%)	5 (17.24%)	13 (52%)	18 (41.86%)	**0.003** ^†^
CMT, μm	228.00 (210.00–253.00)	261.00 (246.00–276.45)	297.00 (268.00–510.00)	282.00 (262.00–329.00)	** <0.001** ^*^
CMV (within 1 mm), mm^3^	0.18 (0.17–0.20)	0.21 (0.19–0.22)	0.23 (0.21–0.40)	0.22 (0.21–0.26)	** <0.001** ^*^
CMV (within 3 mm), mm^3^	2.09(1.92–2.24)	2.29 (2.22–2.34)	2.71 (2.42–3.30)	2.50 (2.22–2.71)	** <0.001** ^*^
CMV (within 5 mm), mm^3^	5.82(5.29–6.12)	6.27 (6.01–6.41)	7.51 (6.48–8.82)	7.10 (6.14–7.25)	** <0.001** ^*^
MA count (panretina), *n*	64.00 (8.00–93.00)	222.00 (163.67–284.00)	338.00 (295.00–390.00)	284.00 (250.00–339.00)	** <0.001** ^*^
MA count (PoA), *n*	41.00 (7.00–64.00)	162.00 (106.00–207.00)	257.00 (191.00–316.00)	195.00 (145.00–214.49)	** <0.001** ^*^
MA count (MPA), *n*	19.00 (0.00–25.00)	55.00 (32.00–88.00)	89.00 (49.00–126.00)	91.00 (62.00–105.00)	** <0.001** ^*^
MA count (FPA), *n*	2.00 (1.00–7.00)	11.00 (4.00–17.00)	14.00 (6.50–32.00)	17.00 (8.0–22.00)	**0.006** ^*^
ISI (panretina), %	0.00 (0.00–0.00)	0.93 (0.44–1.87)	3.47 (1.06–4.29)	4.21 (2.52–6.62)	** <0.001** ^*^
ISI (PoA), %	0.00 (0.00–0.00)	1.28 (0.79–2.63)	3.60 (1.69–9.83)	7.72 (5.48–9.77)	** <0.001** ^*^
ISI (MPA), %	0.00 (0.00–0.00)	0.53 (0.19–1.04)	1.67 (0.28–4.16)	3.35 (1.95–5.98)	** <0.001** ^*^
ISI (FPA), %	0.00 (0.00–0.00)	0.00 (0.00–0.00)	0.00 (0.00–1.58)	0.23 (0.00–1.26)	**0.004** ^*^
Leakage index (panretina), %	0.52 (0.13–1.00)	4.08 (3.00–5.84)	7.90 (3.30–9.52)	8.65 (6.71–11.01)	** <0.001** ^*^
Leakage index (PoA), %	1.60 (0.37–2.19)	9.38 (6.82–14.09)	17.19 (10.36–21.09)	20.41 (13.06–23.37)	** <0.001** ^*^
Leakage index (MPA), %	0.004 (0.00–0.24)	1.66 (0.66–2.47)	2.13 (1.12–4.35)	4.19 (2.53–5.99)	**<** 0.001^*^
Leakage index (FPA), %	0.014 (0.00–0.38)	1.04 (0.04–1.83)	0.77 (0.01–1.99)	1.23 (0.63–2.61)	**0.006** ^*^

*NPDR, nonproliferative diabetic retinopathy; PDR, proliferative diabetic retinopathy; DME, diabetic macular edema; CMT, central macular thickness; CMV, central macular volume; MA count, microaneurysm count; ISI, ischemic index; PoA, posterior area; MPA, mid-periphery area; FPA, far-periphery area*.

*All quantitative parameters are expressed as median (95% CI)*.

* *p value determined by Kruskal–Wallis H test*;

† *p-value determined by chi-squared test. Bold font indicates statistical significance*.

### Correlation Between Panretinal and Regional UWFFA Characteristics and DR Severity

**Figure 2:**
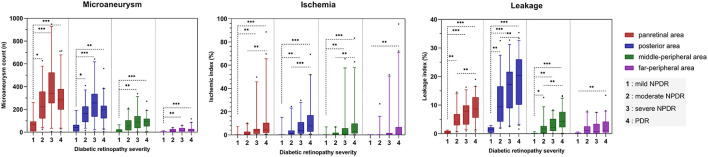
Bar chart showing the relationship and trend between three quantitative UWFFA parameters and DR severity for panretina and each subregion. The bars are expressed as the median ± 95% CI. ^*^p < 0.05, ^**^p < 0.01, ^***^p < 0.001.

**Figure 3:**
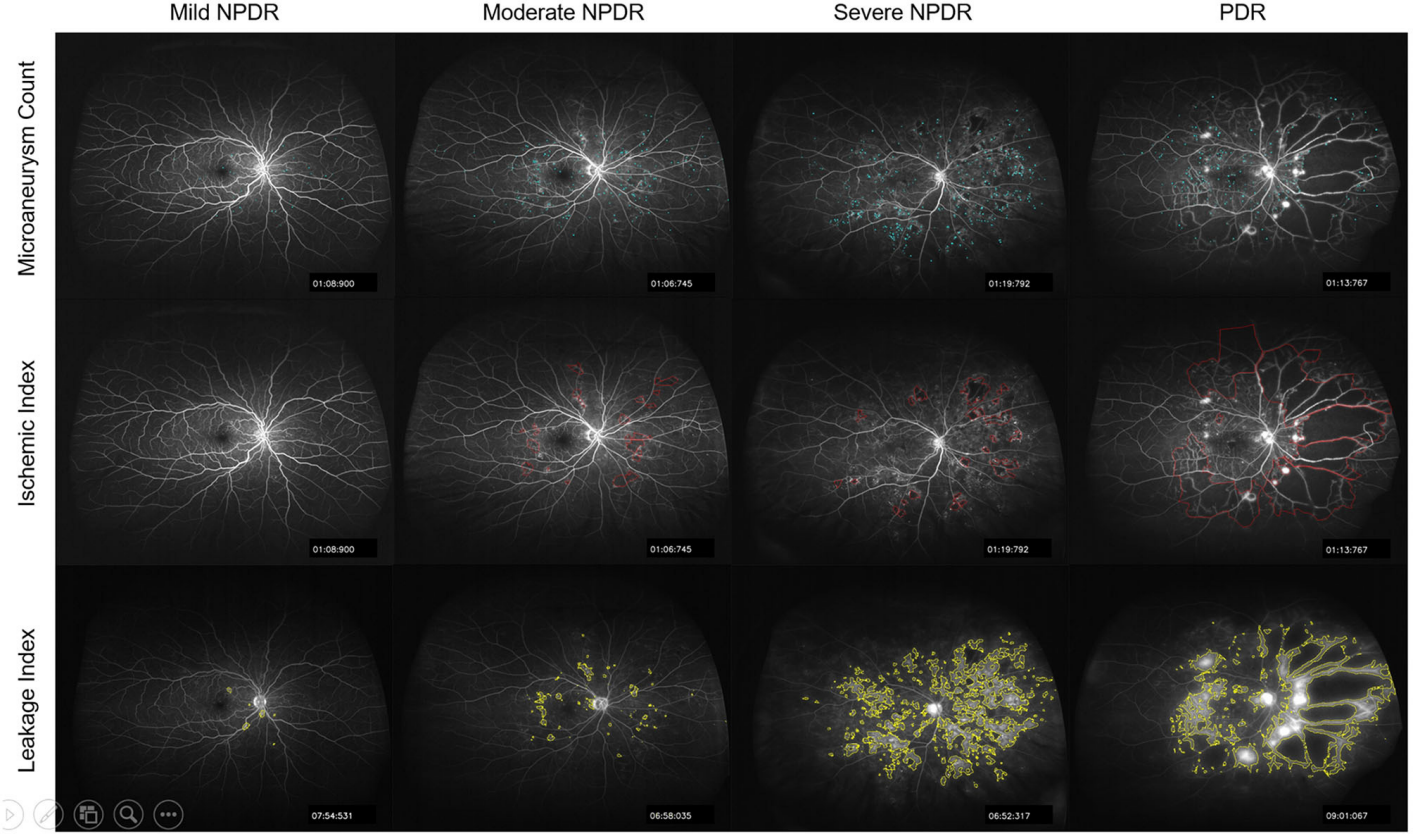
Representative segmented images of microaneurysms, ischemia, and leakage on UWFFA images of patients with different severity of DR.

As the severity of DR increased, panretinal and subregion-specific ISI and leakage index tended to increase (Figures 2, 3). MA count in the NPDR group gradually increased with increasing disease severity, but it was lower in the PDR group than in the severe NPDR group. However, this difference was not statistically significant (all *p* > 0.05). Meanwhile, the topographic analysis showed that all three parameters were higher in the PoA than in the MPA and FPA (all *p* < 0.01). The differences in UWFFA parameters between different DR groups with different severity levels were more pronounced in the PoA (Figure 2).

Multivariate ordinal logistic regression analysis showed that panretinal leakage index and ISI were associated with DR severity, while MA count was not (*p* = 0.000, 0.027, and 0.106, respectively), and posterior leakage index, ISI, and MA count were all associated with DR severity (*p* = 0.026, 0.030, and 0.002, respectively).

### Comparison of UWFFA Characteristics of Eyes With and Without DME

In all the studied eyes, panretinal MA count and leakage index were significantly higher in eyes with DME than those without DME (*p* = 0.044 and 0.001, respectively). However, leakage index, ISI, and MA count in the PoA were significantly higher in eyes with DME than those without DME (*p* < 0.001, 0.022, and 0.012, respectively). Notably, there were no significant differences in any of the parameters between the two groups in the mid- and far-peripheral regions (Table 2).

**Table 2:** Comparison of clinical characteristics between eyes with and without DME.

**Characteristic**	**DME present (*n* = 36)**	**DME absent (*n* = 72)**	***p-*value**
Age, y	60.50 (56.00–64.00)	56.00 (55.00–58.00)	-
Female, *n* (%)	17 (47.22%)	34 (47.22)	-
Right eye, *n* (%)	18 (50%)	18 (50%)	-
CMT, μm	428.00 (397.00–502.00)	255.00 (248.51–262.00)	** <0.001** ^*^
CMV (within 1 mm), mm^3^	0.34 (0.31–0.39)	0.20 (0.19–0.21)	** <0.001** ^*^
CMV (within 3 mm), mm^3^	3.19 (2.90–3.48)	2.22 (2.19–2.28)	** <0.001** ^*^
CMV (within 5 mm), mm^3^	8.66 (7.77–8.99)	6.11 (5.97–6.25)	** <0.001** ^*^
MA count (panretina), *n*	318.00 (262.00–356.50)	252.00 (169.00–284.00)	**0.044** ^*^
MA count (PoA), *n*	213.50 (177.00–237.00)	152.50 (114.00–191.00)	**0.012** ^*^
MA count (MPA), *n*	72.50 (61.00–105.89)	57.50 (39.00–89.00)	0.149^*^
MA count (FPA), *n*	12.00 (7.00–20.00)	10.50 (7.00–17.00)	0.977^*^
ISI (panretina), %	2.79 (1.59–4.48)	1.90 (0.99–3.53)	0.118^*^
ISI (PoA), %	5.10 (2.36–10.05)	2.65 (1.32–4.41)	**0.022** ^*^
ISI (MPA), %	1.69 (0.49–2.91)	1.22 (0.50–2.71)	0.590^*^
ISI (FPA), %	0.00 (0.00–0.24)	0.00 (0.00–0.17)	0.595^*^
Leakage index (panretina), %	8.90 (7.90–11.85)	4.53 (3.05–5.76)	** <0.001** ^*^
Leakage index (PoA), %	21.22 (20.20–24.78)	9.87 (7.11–11.96)	** <0.001** ^*^
Leakage index (MPA), %	3.66 (2.43–5.05)	1.65 (0.96–2.31)	**0.004** ^*^
Leakage index (FPA), %	1.42 (0.51–2.97)	0.64 (0.25–1.04)	0.077^*^

*DME, diabetic macular edema; CMT, central macular thickness; CMV, central macular volume; MA count, microaneurysm count; ISI, ischemic index; PoA, posterior area; MPA, mid-periphery area; FPA, far-periphery area*.

*All quantitative parameters are expressed as median (95% CI)*.

* *p-value determined by Mann–Whitney U-test. Bold font indicates statistical significance*.

Given that the MA count of PDR may be affected by the large NPA, and that there were many neovascularization components in the retinal vascular leakage, we investigated the differences in UWFFA characteristics of eyes with and without DME in NPDR and PDR groups. In patients with NPDR, the panretinal and PoA-specific leakage index, ISI, and MA count were higher in eyes with DME than those without DME (all *p* < 0.05). Multiple linear regression analysis showed that only the leakage index was associated with CMT in the panretina and PoA (*p* = 0.006 and 0.001, respectively). In patients with PDR, only panretinal, PoA-specific, and MPA-specific leakage indexes were significantly different between eyes with and without DME (all *p* < 0.05), and no significant differences were found between the other regional UWFFA parameters. Multiple linear regression analysis showed that neither panretinal nor regional UWFFA parameters were correlated with CMT (all *p* > 0.05).

### Comparison of UWFFA Characteristics of Eyes With and Without PDR

The MA count in the MPA was significantly higher in eyes with PDR than those without PDR (*p* = 0.041). Leakage index and ISI were significantly higher in eyes with PDR than those without PDR, both in panretina and subregion-specific measurements (all *p* < 0.05, Table 3). No significant differences were found between the two groups for any other regional parameters (all *p* > 0.05).

**Table 3:** Comparison of clinical characteristics between eyes with and without PDR.

**Characteristic**	**PDR present (*n* = 43)**	**PDR absent (*n* = 65)**	***p* value**
Age, y	55.00 (51.00–57.00)	58.00 (56.00–61.50)	-
Female, *n* (%)	19 (44.19%)	32 (49.23%)	-
Right eye, *n* (%)	20 (46.51)	34 (52.31)	-
CMT, μm	282.00 (262.00–310.00)	266.00 (259.00–283.00)	0.361
CMV (within 1 mm), mm^3^	0.22 (0.21–0.24)	0.21 (0.21–0.22)	0.350
CMV (within 3 mm), mm^3^	2.50 (2.22–2.67)	2.32 (2.27–2.39)	0.770
CMV (within 5 mm), mm^3^	7.07 (6.16–7.25)	6.38 (6.14–6.49)	0.516
MA count (panretina), *n*	284.00 (250.00–339.00)	260.00 (175.00–317.87)	0.222
MA count (PoA), *n*	195.00 (141.54–215.00)	168.00 (114.00–207.00)	0.609
MA count (MPA), *n*	91.00 (62.00–105.00)	55.00 (35.00–69.00)	**0.041**
MA count (FPA), *n*	17.00 (8.00–22.00)	8.00 (5.50–14.00)	0.087
ISI (panretina), %	4.21 (2.52–6.62)	1.06 (0.44–1.87)	** <0.001**
ISI (PoA), %	7.72 (5.48–10.29)	1.68 (0.94–2.69)	** <0.001**
ISI (MPA), %	3.35 (1.94–6.01)	0.48 (0.18–1.04)	** <0.001**
ISI (FPA), %	0.23 (0.00–1.26)	0.00 (0.00–0.00)	**0.006**
Leakage index (panretina), %	8.65 (6.71–11.28)	3.76 (3.06–5.62)	** <0.001**
Leakage index (PoA), %	20.41 (12.51–23.30)	9.38 (7.10–13.47)	** <0.001**
Leakage index (MPA), %	4.19 (2.63–6.6.09)	1.12 (0.66–2.17)	** <0.001**
Leakage index (FPA), %	1.23 (0.63–2.62)	0.45 (0.04–1.04)	**0.014**

*PDR, proliferative diabetic retinopathy; CMT, central macular thickness; CMV, central macular volume; MA count, microaneurysm count; ISI, ischemic index; PoA, posterior area; MPA, mid-periphery area; FPA, far-periphery area*.

All quantitative parameters are expressed as median (95% CI). ^*^p-value determined by Mann–Whitney U-test. Bold font indicates statistical significance.

### Assessment of the Indexes Most Associated With the Two Adverse Outcomes Using ROC Curves

**Figure 4:**
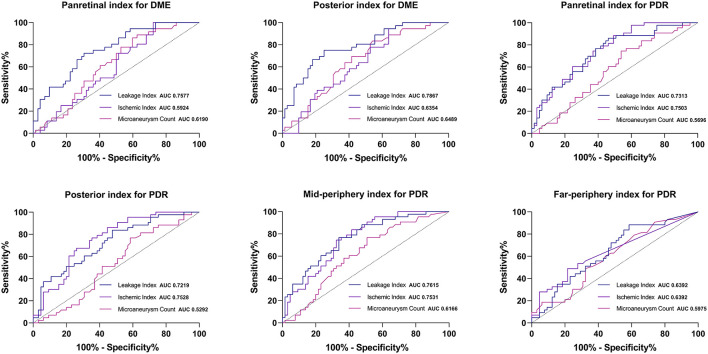
Receiver operating characteristic curve of the regional UWFFA parameters for determining the occurrence of DME and PDR.

Since there were no significant differences in UWFFA parameters in the MPA and FPA between eyes with and without DME, we evaluated ROC curves of the three parameters in the panretina and PoA for predicting DME (Figure 4). For both panretinal and PoA-specific analyses, the leakage index had a higher diagnostic value for DME than ISI and MA count (all *p* < 0.05). ROC curves showed that the leakage index in the PoA had the largest AUC for the determination of DME. The cutoff value at this point was 16.704%, and its specificity and sensitivity for determining the occurrence of DME were both 75%. In addition, multiple linear regression analysis showed that among all the posterior parameters, only leakage index was significantly correlated with CMT and CMV (all *p* < 0.001), and had the highest correlation with CMV in the 5-mm range and the lowest correlation with CMV in the 1-mm range or CMT.

As for PDR, the AUC of ISI was larger than that of leakage index in both panretina and PoA-specific measurements (Figure 4). In the MPA, the AUC of leakage index was larger than that of ISI. Notably, the diagnostic value of MA count for PDR was lower than that of ISI and leakage index, but there was no significant difference in the diagnostic value of ISI and leakage index in any region (all *p* < 0.05, method of Delong et al., 1988). The optimal cutoff values for judging DME and PDR for different UWFFA indexes in different regions are presented in Tables 4, 5.

**Table 4:** Optimal cut-off values and coordinates of the ROC curve for determining the presence of DME.

**Characteristic**	**Cut-off values**	**Sensitivity%**	**Specificity%**	**Youden index**
MA count (panretina), *n*	>153	88.89	37.50	0.2639
MA count (PoA), *n*	>129	83.33	45.83	0.2917
ISI (panretina), %	>0.34	100.00	27.78	0.2778
ISI (PoA), %	>1.01	94.44	36.11	0.3056
Leakage index (panretina), %	>6.709	72.22	69.44	0.4167
Leakage index (PoA), %	>16.704	75.00	75.00	0.5000

*MA count, microaneurysm count; ISI, ischemic index; PoA, posterior area*.

**Table 5:** Optimal cut-off values and coordinates of the ROC curve for determining the presence of PDR.

**Characteristic**	**Criterion values**	**Sensitivity%**	**Specificity%**	**Youden index**
MA count (panretina), *n*	>222	74.42	46.15	0.2057
MA count (PoA), *n*	>119	76.74	41.54	0.1828
MA count (MPA), *n*	>50	76.74	49.23	0.2597
MA count (FPA), *n*	>5	79.07	38.46	0.1753
ISI (panretina), %	>1.058	88.37	50.77	0.3914
ISI (PoA), %	>1.7	86.05	55.38	0.4143
ISI (MPA), %	>0.64	83.72	58.46	0.4218
ISI (FPA), %	>0.325	48.84	78.46	0.2730
Leakage index (panretina), %	>4.173	86.05	53.85	0.3989
Leakage index (PoA), %	>9.393	83.72	52.31	0.3603
Leakage index (MPA), %	>2.233	76.74	66.15	0.4290
Leakage index (FPA), %	>0.044	88.37	41.54	0.2991

*MA count, microaneurysm count; ISI, ischemic index; PoA, posterior area; MPA, mid-periphery area; FPA, far-periphery area*.

## Discussion

In this study, we quantified MA count, ISI, and leakage index parameters in different regions of the retina based on UWFFA images, and analyzed the correlations between these parameters and DR severity. We also evaluated the association of these parameters with DME and PDR, both of which are severe vision-threatening endpoints in patients with DR. Previous studies have demonstrated the correlation of the above three indexes with DR severity (20). Our study has validated some of their conclusions, and meanwhile provides some new findings. Although these three parameters were higher in the eyes with DME than those without DME, the leakage index was more valuable than ISI and MA count in predicting DME. Leakage index, ISI, and MA count in the PoA were more strongly correlated with DR grade and DME occurrence than when they were measured in the panretina, and even less association was observed when they were measured in the mid- and far-periphery. ISI and leakage index, but not MA count, were powerful predictors of PDR. In addition, leakage index was better correlated with retinal volume in the 5-mm perimacular region than in the CMT or 1-mm perimacular region, suggesting that the posterior leakage index better reflects diffuse macular thickening.

Ischemia, leakage, and MA are typical fluorescein angiography features of patients with DR. The availability of UWFFA devices has enabled visualization of the peripheral retina, which was previously limited by conventional devices. Silva et al. (8) first reported that NPA and ISI on UWFFA are associated with DR severity. Ehlers et al. (12, 21) developed a semiautomatic quantitative platform for analysis of ischemia, leakage, and MA on UWFFA images, which they used to systematically evaluate the correlation of these three parameters with DR severity (20). Consistent with their results, our study showed a positive correlation between panretinal leakage index and ISI and DR severity (20). However, we found that panretinal MA count increased with increasing disease severity in the mild, moderate, and severe NPDR groups, but patients in the PDR group had lower panretinal MA count than patients with NPDR, and only MA count in the PoA was significantly correlated with DR severity. NPA in DR eyes starts in the perifoveal retina (22), and as the disease progresses, NPA may also appear in the paramacular or even peripheral retina. The extent of retinal capillary occlusion and NPA make it impossible for MAs to exist alone, and thus panretinal MA count in PDR is reduced instead. Due to the existence of significant ischemia and hypoxia in the PDR eyes, numerous MAs could still be present in the capillary perfusion areas of the PoA. The variability among the involved patients may be the reason for these different results. In addition, it is worth noting that the three parameters measured in the PoA are more meaningful than the panretinal measurements, even though more lesions may be found throughout the retina.

The mechanism of DME formation is complex and multiple factors are involved. VEGF is a potent vasopermeability factor (23), and its expression in the retina is upregulated in response to ischemia/hypoxia (24, 25). With increasing retinal vascular permeability and endothelial cell proliferation (24, 25), the breakdown of the blood-retinal barrier results in retinal edema. Previous studies showed that quantitative UWFA imaging parameters are correlated with concentrations of various aqueous cytokines, namely, VEGF and IL-6 (26). The advent of fluorescence angiography can help us observe the process of leakage of fluid from the blood vessels into tissues, and quantify the severity of leakage through fluorescence angiography images, thus facilitating our exploration of the disease.

Previous studies showed that the occurrence and severity of DME are influenced by both leakage index and MA count in the PoA (16). Our study found that only the posterior leakage index correlated with the severity of DME. Therefore, the leakage index is more important in determining the occurrence of DME than MA. Furthermore, the efficacy of the panretinal leakage index in determining DME was lower than that in the PoA, probably because posterior leakage is more likely to diffuse into the macula and contributes to the occurrence of DME than leakage in the MPA and FPA.

Retina leakage can be derived from multiple sources. Xue et al. (4) classified the causes of leakage into three types based on FFA presentation: MA-driven, peripheral ischemia, and neovascularization. We found that in patients with NPDR, MA count in PoA was significantly higher in eyes with DME than those without DME. Previous studies found a close relationship between leakage and MA (16). The leakage index has been demonstrated as the most sensitive predictor of DME. Therefore, we speculate that the presence of DME in patients with NPDR is more likely to be associated with MA-related leakage. However, in patients with PDR with reduced MA count, there were still several eyes presenting with DME because of severe leakage. Therefore, we suggest that there may be different mechanisms for the occurrence of DME in NPDR and patients with PDR and that the leaky component in patients with PDR is more likely to be of neovascular origin.

Furthermore, in NPDR eyes, the occurrence of DME was associated with ISI, but only in the PoA. The same relationship was not found in PDR eyes. In addition, no clear correlation was found between ISI and the severity of DME, in agreement with Wessel et al. (27) and Fan et al. (28).

The most important finding of this study was to explore the strongest correlations with DME and PDR from the current indexes. We found that the leakage index is optimal in the ROC curves of all three, with high accuracy in disease prediction. Based on the pathogenesis of the retinal vascular disease, MA, ischemia, or neovascularization will eventually all be accompanied by leakage in the fundus, and the leakage index may be the result of a combination of other factors. In the future, the leakage index may become an important index for assessing the severity of fundus vascular diseases.

Both leakage index and ISI are of high value for the diagnosis of PDR, and they are both superior to MA count. The occurrence of PDR can be caused by intraocular tissue hypoxia, which induces elevated levels of VEGF and other proangiogenic factors and promotes neovascularization (29). It has been reported that NPA reflects the severity of fundus hypoxia, and it may correlate with intraocular VEGF levels (5). Therefore, ISI can predict the development of neovascularization or PDR. Conversely, when neovascularization is immature and the blood-retinal barrier is highly permeable, significant leakage can be observed in fluorescence angiography images. Therefore, the leakage index is comparable to the ISI in determining PDR. Changes in these UWFFA parameters after treatment may suggest a greater significance. Babiuch et al. (30) found that patients with PDR underwent a significant reduction in leakage index on UWFFA images of panretina and all subregional areas after anti-VEGF treatment. In addition, it should not be overlooked that MA count also significantly decreased after anti-VEGF treatment (31). Leakage index and MA count may play critical roles in the assessment of treatment outcomes in patients with PDR in the future.

This study has some limitations. First, this was a retrospective observational study with small sample size. Second, peripheral image distortion is a common problem for UWF images and may affect the accuracy of image analysis. Third, only one macula-centered UWFFA image was utilized for analysis, and the complete retinal image puzzle was not acquired using the montage method. Fourth, in our clinical work, we did not set the late images at a specific point in time but within a range, which may cause some degree of variation due to the dynamic nature of fluorescence angiography.

Despite the limitations, our study complements some gaps in the current quantitative study of UWFFA in DR. We explored the in-depth relationship between each of these parameters and two adverse outcomes, PDR and DME, and found the best-evaluated parameter among several commonly used options. We also designed a quantitative method for UWFFA that can be implemented by open software, which will help improve accessibility and enrich the study of UWF images. In addition, the above limitations include common technical problems faced by current UWF image research, such as peripheral aberrations and montage. Subsequently, we expect that the development of technology will allow us to improve upon our results. In the future, studies with larger sample size and more precise timing of DR UWFFA images are needed.

In conclusion, ISI, leakage index, and MA count in the PoA and panretina were correlated with the severity of DR, especially the posterior parameter. The leakage index was more valuable than ISI and MA count in determining the occurrence of DME. ISI and leakage index were of higher value in determining PDR.

## Data Availability Statement

The raw data supporting the conclusions of this article will be made available by the authors, without undue reservation.

## Ethics Statement

The studies involving human participants were reviewed and approved by Clinical Research Ethics Committee of Renmin Hospital of Wuhan University. Written informed consent for participation was not required for this study in accordance with the national legislation and the institutional requirements.

## Author Contributions

GS, XW, and CC: conception, design, and manuscript preparation. GS, XW, and JJ: image processing design. GS, XW, JJ, ZY, MF, XY, and HZ: image and data collection. HZ and CC: manuscript review and revision. All authors read and approved the final manuscript.

## Conflict of Interest

The authors declare that the research was conducted in the absence of any commercial or financial relationships that could be construed as a potential conflict of interest.

## Publisher's Note

All claims expressed in this article are solely those of the authors and do not necessarily represent those of their affiliated organizations, or those of the publisher, the editors and the reviewers. Any product that may be evaluated in this article, or claim that may be made by its manufacturer, is not guaranteed or endorsed by the publisher.
